# Personality traits, pain perception, and patient attitudes toward orthodontic treatment with fixed appliances

**DOI:** 10.3389/fneur.2025.1547095

**Published:** 2025-03-07

**Authors:** Monika Lorek, Anna Jarząbek, Magdalena Sycińska-Dziarnowska, Sylwia Gołąb, Ewa Cichocka, Gianrico Spagnuolo, Krzysztof Woźniak, Liliana Szyszka-Sommerfeld

**Affiliations:** ^1^Private Clinic HaLo Ortho, Gniezno, Poland; ^2^Laboratory of Paediatric Dentistry, Pomeranian Medical University in Szczecin, Szczecin, Poland; ^3^Department of Maxillofacial Orthopaedics and Orthodontics, Pomeranian Medical University in Szczecin, Szczecin, Poland; ^4^Department of Economics and Accounting, West Pomeranian University of Technology in Szczecin, Szczecin, Poland; ^5^University Clinical Hospital no. 2 Pomeranian Medical University in Szczecin, Szczecin, Poland; ^6^Department of Neurosciences, Reproductive and Odontostomatological Sciences, University of Naples “Federico II”, Napoli, Italy; ^7^School of Dentistry, College of Dental Medicine, Kaohsiung Medical University, Kaohsiung, Taiwan; ^8^Laboratory for Propaedeutics of Orthodontics and Facial Congenital Defects, Pomeranian Medical University in Szczecin, Szczecin, Poland

**Keywords:** orofacial pain, orthodontic pain, pain intensity, pain perception, pressure pain threshold, personality traits, stomatognathic system, attitude

## Abstract

**Background:**

Orthodontic pain is defined as orofacial pain induced by orthodontic tooth movement. The application of orthodontic forces activates periodontal sensory receptors, resulting in a cascade of nociceptive pain processing and transduction in both the central and peripheral nervous systems, which is eventually felt by patients. The purpose of this study was to evaluate the association between pain perception, pressure pain threshold (PPT), attitude toward orthodontic treatment, and personality traits in adolescents treated with fixed orthodontic appliances.

**Methods:**

The study involved 60 subjects aged 16 to 18 year-olds divided into 2 groups: group 1 consisted of 30 patients undergoing orthodontic treatment with fixed orthodontic appliances, and group 2 consisted of 30 untreated subjects. The tool for data collection was a questionnaire that assessed pain experience for treated subjects, pain expectation for untreated subjects, and attitude toward treatment using a visual analog scale (VAS) marked at 10-mm intervals. The assessment of patients’ personality profiles was carried out using the NEO Five-Factor Inventory (NEO-FFI). PPT was measured using a digital force algometer. The results were subjected to statistical analysis. The significance threshold was established at *p* < 0.05.

**Results:**

The multivariate analysis showed that treatment status was the only variable affecting patients’ average attitude scores and average pain experience/expectation scores measured using a VAS, and average PPT scores (*p* < 0.0001). Gender and personality traits did not affect PPT scores, pain intensity, and attitude toward treatment (*p* > 0.05). The results of the follow-up univariate analysis demonstrated a significant difference in the patients’ average attitude toward treatment (*p* = 0.017) and PPT scores (*p* < 0.0001) between the treated and untreated groups.

**Conclusion:**

Orthodontic treatment may impact the pressure pain thresholds measured using algometry and patient attitudes toward treatment. This knowledge is essential for orthodontists and patients, as the success of orthodontic treatment largely depends on the patient’s cooperation and motivation, which may be affected by patient’s attitude toward treatment and pain perception. This, in turn, encourages the search for effective methods of pain reduction during orthodontic treatment and attention to communication between orthodontists and patients for a good understanding of the procedures used.

## 1 Introduction

According to the International Association for the Study of Pain (IASP), pain is defined as “an unpleasant sensory and emotional experience associated with, or resembling that associated with, actual or potential tissue damage” (1). Orthodontic pain is an orofacial pain (OFP) induced by orthodontic tooth movement. It can be caused by procedures such as inserting separators, activating and positioning orthodontic archwires, or applying orthopedic forces. The application of orthodontic forces activates periodontal sensory receptors, resulting in a cascade of nociceptive pain processing and transduction in both the central and peripheral nervous systems, which is eventually felt by patients. According to the literature, the incidence of pain accompanying orthodontic treatment with fixed braces can be exceptionally high, affecting up to 90–95% of patients (2–7). Nonetheless, pain perception is highly subjective, with notable inter-individual variability. The level of pain perception depends on the individual pain threshold of each patient, and can be influenced by a number of both external and internal factors, such as age (5, 6, 8–11), gender (8, 9), amount of force applied (12), emotional aspects (13), physical activity levels (14), cultural (15) and genetic (16) factors, past pain experiences (17), etc. (18–24). Previous memories of pain or fear of pain have been shown to intensify the experience of discomfort related to orthodontic treatment, while patients with high personal perceptions of the severity of their malocclusion exhibit high compliance and low levels of pain and discomfort (17). The intensity of pain is the most assessed dimension and can be measured with different instruments. The visual analog scale (VAS), numeric rating scale (NRS), and verbal descriptor scales are the most commonly used self-reported pain perception scales (25, 26). Nevertheless, pain provocation tests using an algometer can also measure pain intensity through pressure trigger points (27, 28).

Orthodontic therapy can be affected by the practitioner’s aptitude and attitude, as well as the perception of pain, personality features, and patient participation. With proper engagement, well-cooperative patients can achieve the best therapeutic outcomes. In contrast, lack of cooperation can cause delays in therapy delays and increase the number of visit. Therefore, the patient’s attitude toward orthodontic treatment plays a significant role in achieving a successful outcome. Moreover, it has been proven that pain can be one of the factors discouraging patients from orthodontic therapy (7, 29–34). In addition, several authors have reported an association between patient’s personality traits and various factors associated with orthodontic treatment, such as pain perception, treatment attitudes, motivation, and/or post-treatment satisfaction (5, 7, 21, 34–39). However, the findings of some studies remain inconclusive (31). Personality traits are common patterns of thinking and behaving that can influence behavior, interests, and satisfaction (40, 41). In particular, Singh et al. (34) and Kadu et al. (7) found a strong correlation between pain perception and personality traits, such as neuroticism and conscientiousness, that is, more pain with higher levels of neuroticism and lower levels of conscientiousness. Abu Alhaija et al. (35) observed that after orthodontic treatment, neuroticism scores decreased, while scores for openness, agreeableness, and conscientiousness increased, leading to improved attitudes toward orthodontic treatment. Campos et al. (21) found that individuals dissatisfied with their treatment exhibited more pronounced psychosocial aspects of pain, which was closely related to their motivation level. Cooper-Kazar et al. (36) found that patients who reported higher levels of pain had more narcissistic features. Moreover, Sergl et al. (42) suggested that there was a strong relationship between patient attitude toward orthodontic treatment and discomfort experienced after appliance insertion. Similarly, Abu Alhaija et al. (31) noted that patients with more positive attitudes experienced less pain during orthodontic treatment. In light of the above, understanding the relationship between patient’s personality traits and pain experience during orthodontic treatment is crucial, as patients with some certain personality traits may need simultaneous psychological support throughout their treatment (7, 34). In addition, personality characteristics can affect a patient’s cooperation during treatment and thus may have an impact on the choice of the type of orthodontic therapy (36). During adolescence, personality may be a key mediator of individual differences in the course and treatment responses during orthodontic treatment (9).

Given the high importance of pain perception during orthodontic treatment and the fact, that patients’ personality characteristics appear to influence pain perception and other factors related to orthodontic treatment, further research on this topic is needed. In addition, only a few studies have been conducted on adolescents. Hence, as understanding the association between orthodontic pain and various factors related to its perception could be an important factor affecting the complete success of orthodontic treatment, the present study aimed to evaluate the association between pain intensity, pressure pain threshold (PPT), attitude toward orthodontic treatment and personality traits in adolescents treated with fixed appliances.

## 2 Methods

### 2.1 Study population

The study was approved by resolution KB-006/35/2022 of the Bioethics Committee of the Pomeranian Medical University in Szczecin. All participants and their parents gave their written informed consent for inclusion in the study.

The study involved 60 subjects aged 16 to 18 with complete permanent dentition. Patients were divided into 2 groups:

Group 1–30 patients undergoing orthodontic therapy with fixed appliances in the initial phase of orthodontic treatment. Subjects in this group were selected among patients presenting for orthodontic treatment at the Orthodontics Clinic in Gniezno, Poland between July 2022 and January 2023.

Group 2–30 patients not undergoing orthodontic treatment. Subjects in this group had not experienced any orthodontic treatment before the study. The participants were selected among the secondary school students in Szczecin from May to June 2023.

Inclusion criteria for patients in both groups included permanent dentition, age between 16 and 18 years, and voluntary consent to participate in the study.

Group 1 patients were treated by a single orthodontics specialist according to a similar protocol. Further inclusion criteria for patients undergoing orthodontic therapy included the presence of Class I malocclusion with mild or moderate crowding, indicating the need for treatment with fixed braces. The treatment started with 0.012-in or 0.014-in NiTi archwires. Archwires for leveling were attached with individual elastomeric ligatures. Patients with the presence of genetic or congenital abnormalities, systemic diseases affecting growth and development, missing teeth, extensive prosthetic restorations, gingival and periodontal diseases, temporomandibular joint dysfunctions, chronic pain, received orthodontic treatment in the past, as well as orthognathic patients and those treated at other orthodontic centers, were excluded from participation in the study.

### 2.2 Data collection

The tool for data collection was a questionnaire that assessed pain experience for treated subjects, pain expectation for untreated subjects, and attitude toward orthodontic treatment using a visual analog scale (VAS) marked at 10-mm intervals (7, 31, 34) (Supplementary Files 1 and 2). All subjects were presented with a brief explanation about the aim of this study and clarification of some questions included in the questionnaire and how to score them. Patients were encouraged to ask for help or further explanation if they encountered any difficulty in understanding or scoring the questionnaires.

Each subject’s previous knowledge of orthodontic treatment was assessed by asking whether he or she had any idea about orthodontic treatment (yes/no). The severity of pain was evaluated using a questionnaire consisting of 9 questions regarding self-reported pain (assessment of pain predicted/expected in untreated patients and pain experienced during treatment in patients treated with fixed braces; Supplementary File 1) and assessed using the visual analog scale (VAS) based on a line marked at 10-mm intervals whose ends are anchored and defined with verbal descriptors such as” extremely likely” and” extremely unlikely.” Each patient was asked to mark the line nearest their expectation or experience. The Likert response format was used for all questions. The scores for the 9 questions were averaged to get one score referred to as the average pain perception score. On the VAS line, the lowest scores indicate less pain experienced/expected during orthodontic treatment, and the highest scores indicate more pain experienced/expected. With the help of the VAS scale, the dynamics of changes in pain sensation were assessed in successive time periods of the study. In those treated with fixed braces (Group 1), pain sensation was assessed before brace installation (T0), within 24 h after brace installation (T1), on day 7 (T2), and 4 weeks after brace installation (T3). For each patient, the average pain perception/experience scores measured using a VAS were a mean of pain perception from T0 to T3. In untreated patients (Group 2), the severity of expected pain was assessed once.

Participant attitudes toward orthodontic treatment were analyzed using a questionnaire consisting of 12 questions and assessed using the VAS and Likert scales (Supplementary File 2). Subjects were asked to answer questions by placing a mark on the line nearest to their attitude toward the treatment. On the VAS line, the lowest scores indicate a more positive attitude toward orthodontic treatment, and the highest scores indicate a more negative attitude toward orthodontic treatment. The scores for the 12 questions were averaged to get one score referred to as the average attitude toward treatment score. In patients undergoing orthodontic treatment (Group 1), attitude toward treatment was assessed before brace installation (T0), within 24 h after brace installation (T1), on day 7 (T2), and 4 weeks after brace installation (T3). For each Group 1 patient, the average attitude toward treatment scores measured using a VAS were a mean of attitude from T0 to T3. In untreated patients (Group 2), the attitude toward orthodontic treatment was assessed once.

Patient personality traits were assessed using the NEO Five-Factor Inventory (NEO-FFI)—Polish adaptation (43, 44). This test provides a comprehensive assessment of personality using five major domains: neuroticism (N), extraversion (E), openness (O), agreeableness (A), and conscientiousness (C). The test consists of 60 items, 12 for each domain, and subjects fill in their response to each statement by choosing one of five answers: strongly agree, agree, neutral, disagree, and strongly disagree. Each domain was classified as very high, high, average, low, and very low. For convenience, in statistical analysis, very high and high classes were considered high, and very low and low classes were considered low. This test is recognized for its brevity, reliability, comprehensiveness, and validity in assessing an individual’s personality traits (43–45).

After completing the scoring, each questionnaire was checked to see if all the items were scored, and the subject was asked to score any missed items.

### 2.3 Algometric analysis

The participants’ pressure pain thresholds were assessed using an electronic pressure algometer. The same examiner trained to identify and locate the investigated trigger points evaluated all participants. The trigger points assessed were masseter points situated in the projection of the right and left mandibular angles. The examiner performed the algometer measurements alternately on the right and left sides with a constant sequence of examination. There was a five-second interval between examining the right and left sides. The assessment was performed with the patient’s dental arches in a slightly open position, and the muscles relaxed. During the examination, the footplate of the algometer was always held perpendicular to the skin, in the center of the belly of the examined muscle, applying a constant force of 18 N (pressure 180 kPa) for a duration of 2 s. The patients described the algometry results for the masticatory motor system’s components by selecting the pain intensity each time on a 100 mm Visual Analogue Scale (VAS) ruler. The participants were asked to indicate when the sensation changed from pressure only to pressure and pain using a hand-held switch that recorded the PPT value in the digital algometer when triggered, and at that moment, the pressure was stopped, and the value displayed. The device used in the present study had a button controlled by the patient, who was asked to press it at the beginning of the pain sensation. Before PPT measurements, each patient underwent a short training session to familiarize themselves with the algometer, its hand-held device, and its application method. The measurements were performed twice, with a 20-min interval between each series. The average of the measurements for each point was used for the statistical analysis. In patients undergoing orthodontic therapy (Group 1), the algometric test was performed before the installation of braces (T0), within 24 h after the installation of braces (T1), on day 7 (T2), and 4 weeks after brace installation (T3). For each Group 1 patient, the average PPT scores measured using a VAS scale were derived as a mean of PPT scores from T0 to T3. The algometry test was performed once in patients not undergoing orthodontic treatment (Group 2).

### 2.4 Method error

The reliability of the personality test was tested on all questions using Cronbach’s alpha (46). The Cronbach’s alpha score was 0.84 for the personality test, indicating good internal consistency. Ten subjects answered the questionnaire twice over a 2-week interval. Reliability was carried out on all questions using the correlation coefficient test. The correlation coefficient was high (0.90).

### 2.5 Statistical analysis

The Mann–Whitney U test was used to compare age between sex and treatment groups. The normality was assessed using the Shapiro–Wilk’s test. The multivariate analysis of variance (MANOVA) has been used to seek significant differences in the values of parameters, such as attitude toward treatment, pain intensity measured using the VAS scale, and the pain threshold measured using an algometer between the given groups. The univariate analysis of variance (ANOVA) / Kruskal-Wallis tests have been performed to further determine the parameters presenting a significant difference in the groups deemed significant in MANOVA. The alpha significance levels have been adjusted for multiple testing, with *p*-value <0.05 assumed as the baseline statistical significance level. The calculations have been performed using R statistical software 4.3.2.

## 3 Results

The age range of patients was 16–18, with a median age of 16.95 years (interquartile range—IQR = 16.02–18.5) in the treated group and 18 years (IQR = 17.65–18.07) in the untreated group. There was no difference in age between the treated and untreated groups. Women were significantly younger than men (median age of 17 years, IQR = 16.02–18 vs. median age of 18 years, IQR = 17.3–18.37, respectively). The results of this analysis are presented in Table 1. No significant differences were found in the average attitude scores, average pain perception, and average PPT scores between the subjects with previous knowledge about orthodontic treatment and subjects who did not have previous knowledge about orthodontic treatment in the treated and untreated groups.

**Table 1:** Comparison of age between gender and treated/untreated groups.

Variables		Age	
		Median (IQR)	*p*-value
Gender	Female	17 (16.02–18)	0.026^*^
	Male	18 (17.3–18.37)	
Patient group	Treated	16.95 (16.02–18.5)	0.19
	Untreated	18 (17.65–18.07)	

IQR, interquartile range.

* Statistically significance difference.

Table 2 presents the results of a multivariate analysis (MANOVA) of dependent variables differences, such as average attitude towards treatment scores and average pain experience/expectation scores assessed using a VAS scale, and average PPT scores in different groups of patients divided by treatment status (treated vs. untreated group), sex (females vs. males), and personality parameters (NEO-FFI domains, e.g., high vs. medium vs. low neuroticism etc.). The multivariate analysis only showed a significant difference in the examined variables between the treated and untreated patients (*p* < 0.0001). There were no significant differences in the variables in groups of patients divided based on sex or different NEO-FFI personality traits (*p* > 0.05). Treatment status was the only factor affecting patients’ average PPT scores, pain perception, and attitude toward treatment (Table 2).

**Table 2:** The results of multivariate analysis of variables differences in different groups of patients divided by treated/untreated group, gender, and personality parameters (NEO-FFI domains).

	MANOVA test results for dependent variables: average PPT scores, average attitude towards treatment and average pain perception using a VAS scale	
	Wilk’s test score	*p*-value
Patient group	0.622	< 0.0001^*^
Gender	0.877	0.113
Neuroticism	0.878	0.428
Extraversion	0.906	0.607
Openness to Experience	0.959	0.925
Agreeableness	0.795	0.323
Conscientiousness	0.795	0.104

MANOVA, multivariate analysis of variance; NEO-FFI, NEO Five-Factor Inventory; PPT, pressure pain threshold; VAS, visual analog scale.

* Statistically significance difference.

**Figure 1:**
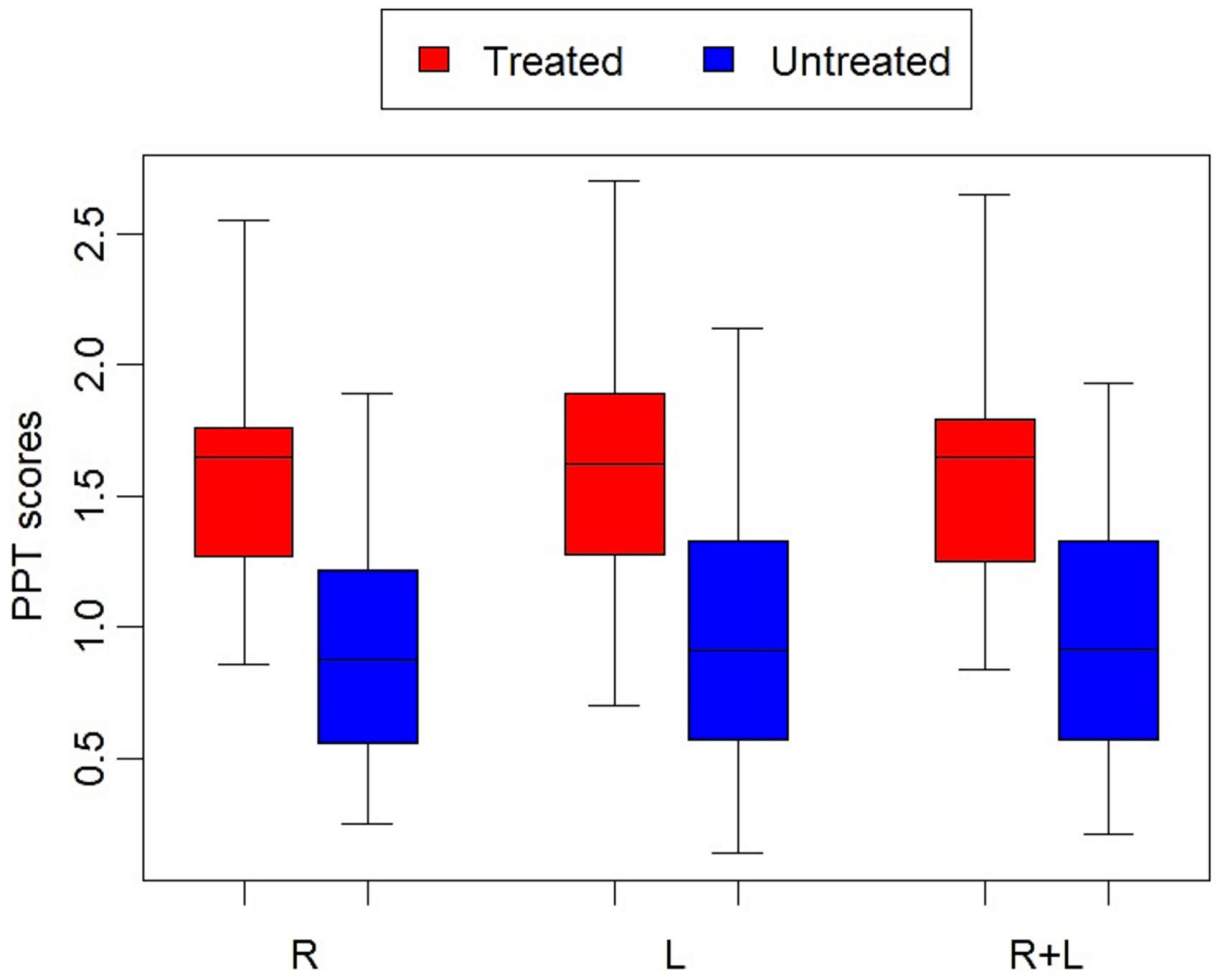
The results of the algometer pain threshold (PPT) scores [right (R), left (L), average R + L] between the treated and untreated groups. Box: Interquartile range, Horizontal line in the Box: Median, Whiskers: Min-Max.

The results of the follow-up univariate analysis demonstrated significant differences in the patients’ attitude towards treatment (*p* = 0.017) (Table 3) and algometer pain thresholds (right, left and average right + left) (*p* < 0.0001) between treated and untreated groups (Table 3, Figure 1). There was no significant difference between treated and untreated subjects in terms of pain intensity assessed using the VAS scale (Table 3). Descriptive analysis showed that average attitude scores and algometer pain threshold scores were higher in patients from the treated group than in the untreated group (Table 4). The average attitude scores and PPT scores did not differ significantly between the three groups (low, medium, and high) within the same personality trait and between girls and boys. Similarly, the average pain perception (experience/expectation) scores did not vary significantly between the three groups (low, medium, and high) within the same personality trait and between girls and boys (Table 4).

**Table 3:** The results of univariate analysis of variables differences between treated and untreated patient groups.

	Univariate ANOVA/Kruskal Wallis test results									
	Attitude towards treatment		Average pain using a VAS scale		Algometer PPT right+left		Algometer PPT right		Algometer PPT left	
	Kruskal Wallis chi squared	*p*-value	*F* value	*p*-value	*F* value	*p*-value	*F* value	*p*-value	*F* value	*p*-value
Patient group	5.677	0.017^*^	0.405	0.527	25.17	<0.0001^*^	26.433	<0.0001^*^	25.138	<0.0001^*^

The analysis was conducted using the ANOVA or Kruskal Wallis tests, depending on the group parameters. ANOVA, univariate analysis of variance; PPT, pressure pain threshold; VAS, visual analog scale.

* Statistically significance difference.

**Table 4:** Descriptive analysis of the investigated parameters in groups of patients based on gender, treated/untreated group, and personality traits.

Variables		Attitute towards treatment		Pain perception on the VAS scale		Algometer PPT					
						Right (R)		Left (L)		Average R + L	
		Mean ± SD	Median (IQR)	Mean ± SD	Median (IQR)	Mean ± SD	Median (IQR)	Mean ± SD	Median (IQR)	Mean ± SD	Median (IQR)
Gender	Female	3.42 ± 0.84	3.42 (2.77–4.05)	4.76 ± 1.91	5 (3.34–5.97)	1.25 ± 0.57	1.23 (0.87–1.72)	1.23 ± 0.54	1.25 (0.87–1.64)	1.23 ± 0.55	1.25 (0.86–1.71)
	Male	3.98 ± 1.25	4 (3.61–4.73)	4.58 ± 2.06	4.68 (3.49–6)	1.27 ± 0.58	1.31 (0.78–1.73)	1.31 ± 0.65	1.31 (0.77–1.73)	1.31 ± 0.59	1.35 (0.92–1.73)
Patient group	Treated	**3.98 ± 1.03**	**4 (3.67–4.61)**	4.51 ± 2.16	4.87 (2.65–5.79)	**1.58** ± **0.42**	**1.65 (1.27–1.76)**	**1.6** ± **0.51**	**1.62 (1.28–1.89)**	**1.59** ± **0.46**	**1.65 (1.25–1.79)**
	Untreated	**3.42** ± **1.10**	**3.46 (2.77–4.12)**	4.83 ± 1.78	4.81 (3.88–6)	**0.95** ± **0.49**	**0.88 (0.56–1.22)**	**0.95** ± **0.49**	**0.91 (0.57–1.33)**	**0.97** ± **0.49**	**0.92 (0.57–1.33)**
Neuroticism	Low	4.17 ± 1.28	4.6 (3.55–4.97)	5.04 ± 1.89	5.12 (4.12–5.90)	1.17 ± 0.62	1.1 (0.64–1.71)	1.23 ± 0.69	1.35 (0.66–1.77)	1.26 ± 0.63	1.4 (0.88–1.74)
	Medium	3.50 ± 1.05	3.67 (2.83–4.22)	4.95 ± 1.96	5 (3.81–6. 5)	1.30 ± 0.42	1.3 (0.99–1.70)	1.29 ± 0.40	1.3 (0.98–1.66)	1.29 ± 0.40	1.28 (1–1.69)
	High	3.70 ± 1.03	3.75 (3.42–4.17)	4.14 ± 2.00	3.88 (2.65–5.56)	1.26 ± 0.70	1.17 (0.70–0.68)	1.26 ± 0.75	1.15 (0.59–1.63)	1.26 ± 0.72	1.14 (0.63–1.71)
Extraversion	Low	3.71 ± 0.94	3.67 (3.21–4.22)	4.24 ± 2.11	3.87 (2.37–5.24)	1.20 ± 0.71	1.1 (0.59–1.75)	1.24 ± 0.77	1.1 (0.53–1.81)	1.22 ± 0.73	1.1 (0.53–1.81)
	Medium	3.74 ± 1.14	3.9 (3–4.6)	4.69 ± 1.88	5 (3.87–5.62)	1.24 ± 0.51	1.3 (0.89–1.7)	1.26 ± 0.54	1.28 (0.87–1.62)	1.28 ± 0.50	1.28 (0.93–1.66)
	High	3.63 ± 1.19	3.67 (3.31–4.21)	5.16 ± 1.93	5.87 (4.53–6.12)	1.36 ± 0.46	1.63 (0.95–1.73)	1.32 ± 0.47	1.39 (0.93–1.74)	1.33 ± 0.45	1.6 (0.96-1.73)
Openness to Experience	Low	3.68 ± 1.09	3.83 (2.94–4.25)	4.45 ± 2.01	4.43 (2.71–5.71)	1.31 ± 0.45	1.27 (1.07–1.74)	1.25 ± 0.40	1.30 (0.99–1.57)	1.28 ± 0.40	1.30 (1.03–1.48)
	Medium	3.57 ± 1.22	3.63 (2.87–4.38)	4.46 ± 1.99	4.68 (3.43–5.34)	1.25 ± 0.58	2.33 (0.86–1.70)	1.26 ± 0.66	1.25 (0.73–1.74)	1.28 ± 0.60	1.25 (0.88–1.74)
	High	4.02 ± 0.77	3.95 (3.48–4.38)	5.36 ± 1.84	6 (4.14–6.68)	1.25 ± 0.71	1.34 (0.64–1.76)	1.32 ± 0.69	1.33 (0.86–1.80)	1.28 ± 0.69	1.30 (0.71–1.77)
Agreeableness	Low	3.71 ± 0.59	3.90 (3.42–4.20)	5.14 ± 1.93	4.75 (3.87–6)	1.38 ± 0.43	1.37 (1.01–1.73)	1.03 ± 0.26	1 (1–1)	1.19 ± 0.31	1 (1–1.36)
	Medium	3.53 ± 1.09	3.67 (2.88–4.18)	4.37 ± 2.05	4 (2.69–6)	1.33 ± 0.58	1.24 (0.93–1.78)	1.34 ± 0.59	1.43 (0.92–1.72)	1.36 ± 0.56	1.40 (0.97–1.76)
	High	3.87 ± 1.17	3.80 (3.42–4.75)	4.88 ± 1.93	5.19 (3.98–5.85)	1.18 ± 0.59	1.22 (0.62–1.67)	1.25 ± 0.65	1.24 (0.66–1.67)	1.21 ± 0.61	1.25 (0.61–1.73)
Conscientious-ness	Low	3.70 ± 1.18	3.75 (3.14–3.97)	3.92 ± 0.94	3.79 (3.18–4.67)	1.19 ± 0.68	1.18 (0.68–1.53)	1.20 ± 0.67	1.11 (0.70–1.57)	1.22 ± 0.68	1.10 (0.65–1.68)
	Medium	5.15 ± 1.91	5.37 (5.25–6.50)	3.91 ± 0.91	3.83 (3.50–4.42)	1.29 ± 0.64	1.20 (0.86–1.80)	1.26 ± 0.61	1.25 (0.84–1.75)	1.25 ± 0.60	1.20 (0.86–1.73)
	High	4.33 ± 2.26	4.62 (3.75–5.38)	3.18 ± 1.37	3.42 (2.60–4.25)	1.29 ± 0.47	1.32 (0.95–1.63)	1.35 ± 0.43	1.40 (1.15–1.66)	1.32 ± 0.45	1.33 (1.17–1.70)

The differences deemed significant by the MANOVA, and univariate ANOVA analysis were outline in bold font. SD, standard deviation; IQR, interquartile range; PPT, pressure pain threshold; VAS, Visual Analogue Scale.

## 4 Discussion

The present study evaluated the association between orthodontic pain and various factors related to its perception during treatment. For this purpose, we assessed the relationship between pain perception, pressure pain threshold (PPT), attitude toward orthodontic treatment, and personality traits in adolescents aged 16–18 treated orthodontically using fixed appliances. Adolescence is a critical developmental phase characterized by rapid growth and hormonal changes that can affect musculoskeletal health (47). Orthodontic treatment during adolescence is widely considered essential for improving both oral health and quality of life by correcting malocclusions, improving oral function, and promoting psychological well-being (48). During adolescence, personality may be a key mediator of individual differences in the course and treatment responses during orthodontic treatment. This critical stage of adolescent psychological development may make orthodontic treatment an unpleasant and difficult experience. At this stage of development, emotions are amplified, often due to a lack of adequate structuring of coping strategies (21). In light of the above, understanding the relationship between patient’s personality traits and pain experience during orthodontic treatment in this age group is particularly important.

In this examination, we used validated tools to assess the pain experience/expectation, patients’ attitude towards treatment, and personality traits. Pain perception was evaluated utilizing a Visual Analog Scale (VAS) at 10 mm intervals, offering outcomes ranging from extremely likely to extremely unlikely, proving to be a reliable and sensitive measure. The VAS scale is widely used for measuring pain, and it has been described as a reliable, easy, and subjective method of measuring pain intensity with certain advantages over verbal scales (25–28). Personality traits were evaluated using the NEO-FFI test, which effectively measures the five major personality aspects: neuroticism, extraversion, openness, agreeableness, and conscientiousness. This test is recognized for its brevity, reliability, comprehensiveness, and validity in assessing an individual’s personality traits. In addition, it is easy to answer, score, and interpret and is well-documented in the literature (43–45). The participants’ pressure pain thresholds were measured using an electronic pressure algometer. It should be noted that the use of different tools to assess the effects of personality traits on orthodontic patients in previous studies makes comparisons more difficult (49–51). In our study, pain sensation, attitude toward treatment, and pressure algometry in orthodontically treated adolescents were assessed 4 times at the initial stage of the treatment. For each patient, the average pain experience/expectation and attitudes scores were a mean of the assessments from T0 to T3.

In this study, treatment status was the only factor that affected patients’ average PPT scores and attitudes toward treatment. The results showed that treated patients had higher average attitude scores than untreated subjects. Patient attitude plays a major role in the success of treatment. This can be attributed to personal knowledge and information gained from orthodontic experiences. Our findings were consistent with those of Bos et al., who reported an increased positive attitude in treated patients compared to untreated ones (49). However, it should be noted that these authors used a different questionnaire to measure attitudes toward orthodontic treatment. On the other hand, our results disagreed with the findings of Abu Alhaija et al. (31) and Lagerström et al. (50), who found no significant difference in attitudes toward orthodontic treatment between treated and untreated patients. This discrepancy could be attributed to differences in study design, types of orthodontic treatment used, timing of assessments during the study, racial factors, and/or psychosocial considerations. Moreover, studies have shown a significant relationship between orthodontic pain and attitudes toward treatment (7, 31, 34). The study of Abu Alhaija et al. (31) presented that patients with a more positive attitude experienced less pain during orthodontic treatment. In addition, pain perception was lower in patients with prior knowledge about orthodontic treatment, consistent with findings by Touyz and Marchand (51), who suggested that informing patients about expected discomfort can reduce pain during treatment. When comparing study results, it is important to remember that pain experienced during orthodontic treatment is not constant, with an initial increase and a subsequent decrease in pain intensity (6, 52). Our study assessed pain intensity and attitudes toward treatment in orthodontically treated adolescents early in the fixed orthodontic treatment (between the period before treatment and 4 weeks after brace installation). In the study by Abu Alhaija et al. (31), the treated group consisted of adult patients currently undergoing orthodontic treatment or in the retention phase. In their project, the instrument for data collection was a similar questionnaire that included an assessment of patients’ personality profiles (NEO-FFI), pain expectations for untreated subjects, pain experiences for treated subjects, and attitudes toward orthodontic treatment using the VAS (31).

As mentioned earlier, pain intensity can be measured with different instruments. The visual analog scale (VAS), numeric rating scale (NRS), and verbal descriptor scales are the most commonly used self-reported pain perception scales (25, 26). Nevertheless, pressure algometry (PA) can also be used to measure pain intensity through the pressure trigger points for pain (27, 28). PA is a method described to objectify the PPT. According to the literature, PA is a diagnostic test that assesses muscle pain quantitatively and ensures the repeatability of the applied diagnostic factor (53). This technique is a well-known and well-validated method to induce acute experimental pain (54). In our study, PPT was measured using an electronic pressure algometer (pressure device that induces mechanical stimuli) to standardize the amount of pressure applied, similar to that when performing muscle palpation. The results of the study showed that the algometer pain threshold scores significantly differed between patients in the treated and untreated groups. The trigger points assessed were the masseter points located in the projections of the left and right mandibular angles. When interpreting the study results, it should be noted that some authors found only weak correlations between pressure algometry and the subjective reports of pain, such as those measured by scales (28, 55). This may be due to the different nature of the two types of instruments, which measure the same feature but do so in significantly different manners. In this context, it could be noted that pain has been accepted as a sensation influenced by several aspects, so it is hard to determine how much self-reported pain results from local stimulation of the injured site or the presence of an emotional component. This could be in line with our findings, as the results of the univariate analysis demonstrated significant differences in algometer pain thresholds between treated and untreated groups. However, when assessed pain experience/expectation using a VAS scale, no significant difference was found between the pain intensity of the treated and untreated subjects. In contrary, some previous studies found moderate or good correlation between the algometry and the self-reported pain perception scales (56, 57).

In our study, no significant differences in attitude toward orthodontic treatment, pain perception, and PPT scores were detected in any of the five factors of personality traits. The outcome of this study agrees with the results of Abu Alhaija et al. (31), who found no significant correlation between personality traits and pain perception or attitude toward treatment. Similarly, Al-Omiri et al. (38) reported no association between pain levels and personality traits. However, in their study, neuroticism influenced overall treatment satisfaction. Bos et al. (58) concluded that personality traits alone cannot predict patient cooperation during orthodontic treatment. Amado and Sierra (59) also reported no significant associations between psychological characteristics and patient cooperation. These results differ from other studies that found significant correlations between personality traits and factors such as pain perception, treatment attitudes, and patient satisfaction or cooperation (41). The literature demonstrated a relationship between neuroticism and conscientiousness with pain perception during orthodontic treatment (7, 34, 35). Higher levels of neuroticism were previously correlated with higher degrees of pain perception while lower levels of conscientiousness were associated with increased pain perception (7, 34). Similarly, Cooper-Kazar et al. (36) observed that patients who reported higher pain levels also had lower self-esteem regulation and more pronounced narcissistic traits. In turn, other studies found a correlation between anxiety and higher pain perception scores during orthodontic treatment (5, 39).

Similarly, this research found that gender did not affect patients’ PPT scores, pain perception, and attitude toward treatment. Bos et al. (49) and Amado and Sierra (59) also reported that gender is not associated with a patient’s attitude and cooperation during orthodontic treatment. Contrary to our results, Bergius et al. observed higher pain perception in females than males (5). Similar results were observed by Abu Alhaija et al. (31), who found that average pain perception/experience during orthodontic treatment was affected by gender, and females were more sensitive to pain than males. In this context, it should be noted that various bio-physiological and psychosocial factors can contribute to gender differences in pain perception during adolescence. It has been shown that in response to painful stimulus, females have significantly greater activation of the contralateral prefrontal cortex, the contralateral insula and the thalamus compared to males, suggesting an inherent sexual dimorphism in response to pain. In addition, the difference in pain perception among males and females changes significantly after puberty/menarche onset (initiation of menstrual cycles) due to complex central/peripheral interactions between pain specific neurotransmitters and ovarian hormones (9, 60, 61).

In light of the above, in our study the lack of the differences in pain perception and attitude toward treatment with respect to gender and personality traits could be attributed to several factors, such as a relatively small sample size and a specific study population with a narrow age range, in which women were significantly younger than men, which may limit the generalizability of the study results. These factors can make comparisons with other researchers difficult. Taking this into account, future research should consider increasing and balancing the sample size. When interpreting the study results, it should also be taken into consideration the fact that there are some problems with personality measurement in adolescents (62). The problems with factor replicability may be due to developmental changes occurring during this period. Personality traits continue to change throughout adolescence, and the structure and coherence of the five factors vary at different ages (63, 64). In addition, comparisons with other studies are difficult because of differences in study designs, including using different questionnaires to assess patient attitudes toward orthodontic treatment and/or personality characteristics (5, 36, 49, 59). It should also be noted that many other factors, such as racial backgrounds, cultural, social, and demographic factors, may also impact the results of the study. Therefore, further well-designed research is required to evaluate the relationship between pain intensity and other factors related to orthodontic treatment on a larger sample size in different geographic locations and using comprehensive and reliable data collection tools.

## 5 Conclusion

The results of our study, despite its limitations, indicate that in the study population, orthodontic treatment may impact the pressure pain thresholds as tested using algometry and patient attitudes toward treatment. This knowledge is essential for orthodontists and patients as the success of orthodontic treatment largely depends on the patient’s cooperation and motivation, which may be affected by patient’s attitude toward treatment and pain perception. This, in turn, encourages the search for effective methods to reduce pain during orthodontic treatment and to pay attention to communication between orthodontists and patients for a good understanding of the procedures used. It is recommended that psychological assessment of the patient should be given due importance before treatment, during treatment and post treatment in order to maximize patient compliance and improve patient satisfaction.

## Data Availability

The original contributions presented in the study are included in the article/Supplementary material, further inquiries can be directed to the corresponding author.
