# Supplementary material for: Personality traits, pain perception, and patient attitudes toward orthodontic treatment with fixed appliances
Source: Front Neurol. 2025 Mar 7;16:1547095. doi: 10.3389/fneur.2025.1547095 (PMC11927090; doi:10.3389/fneur.2025.1547095)
Supplement: Supplementary file 1 [file Table_1.docx]

**Pain Expectation/Untreated and Pain Experience/Treated Subjects.**

Below you will find questions regarding the expected pain or pain occurring during orthodontic procedures. Please complete the answers based on the scale from 0 to 10 presented below.

1/ Do you think that placing of separators between your teeth is/will be painful?

extremely unlikely 0|__|__|__|__|__|__|__|__|__|__|10 extremely likely

2/Do you think that placing of bands on your posterior teeth is/will be painful?

extremely unlikely 0|__|__|__|__|__|__|__|__|__|__|10 extremely likely

3/Do you think that bond up of brackets causes /caused pain?

extremely unlikely 0|__|__|__|__|__|__|__|__|__|__|10 extremely likely

4/ Do you think that wire changing is/was painful?

extremely unlikely 0|__|__|__|__|__|__|__|__|__|__|10 extremely likely

5/ Do you feel/expect pain from wearing of elastics?

extremely unlikely 0|__|__|__|__|__|__|__|__|__|__|10 extremely likely

6/ Do you feel/expect pain from wearing of head gear?

extremely unlikely 0|__|__|__|__|__|__|__|__|__|__|10 extremely likely

7/ Do you feel/expect pain from wearing of retainers?

extremely unlikely 0|__|__|__|__|__|__|__|__|__|__|10 extremely likely

8/ Do you think that impression taking is/was painful?

extremely unlikely 0|__|__|__|__|__|__|__|__|__|__|10 extremely likely

9/ Did/Do you feel/expect pain during debonding?

extremely unlikely 0|__|__|__|__|__|__|__|__|__|__|10 extremely likely

Thank you.
