# Supplementary material for: Personality traits, pain perception, and patient attitudes toward orthodontic treatment with fixed appliances
Source: Front Neurol. 2025 Mar 7;16:1547095. doi: 10.3389/fneur.2025.1547095 (PMC11927090; doi:10.3389/fneur.2025.1547095)
Supplement: Supplementary file 2 [file Table_2.docx]

**Patient’s Attitude Toward Orthodontic Treatment.**

Below you will find 12 questions related to your attitude to orthodontic treatment. Please answer by marking the appropriate place on the scale shown below.

1/ Braces cause a lot of trouble?

extremely unlikely 0|__|__|__|__|__|__|__|__|__|__|10 extremely likely

2/ When you wear braces, you need to adjust your dietary habits??

extremely unlikely 0|__|__|__|__|__|__|__|__|__|__|10 extremely likely

3/ Orthodontists always say that you have to wear your braces more often than is really necessary?

extremely unlikely 0|__|__|__|__|__|__|__|__|__|__|10 extremely likely

4/ Orthodontic treatment often has no use at all?

extremely unlikely 0|__|__|__|__|__|__|__|__|__|__|10 extremely likely

5/ It is absolutely necessary to care more for your oral hygiene when you are wearing braces?

extremely unlikely 0|__|__|__|__|__|__|__|__|__|__|10 extremely likely

6/ People wearing braces are more often bullied than people without braces?

extremely unlikely 0|__|__|__|__|__|__|__|__|__|__|10 extremely likely

7/It is nonsense visiting an orthodontist after your braces have been removed?

extremely unlikely 0|__|__|__|__|__|__|__|__|__|__|10 extremely likely

8/Elastics which should be worn with braces often have no use?

extremely unlikely 0|__|__|__|__|__|__|__|__|__|__|10 extremely likely

9/ It is not a problem at all when you stop treatment as soon as your teeth are straight?

extremely unlikely 0|__|__|__|__|__|__|__|__|__|__|10 extremely likely

10/ Orthodontists often give indistinct information?

extremely unlikely 0|__|__|__|__|__|__|__|__|__|__|10 extremely likely

11/ Orthodontists always have something to complain about their patients?

extremely unlikely 0|__|__|__|__|__|__|__|__|__|__|10 extremely likely

12/ Orthodontists often spend very little time with their patients?

extremely unlikely 0|__|__|__|__|__|__|__|__|__|__|10 extremely likely

Thank you.
